# Supplementary material for: Effect of Heterogeneous Mixing and Vaccination on the Dynamics of Anthelmintic Resistance: A Nested Model
Source: PLoS One. 2010 May 18;5(5):e10686. doi: 10.1371/journal.pone.0010686 (PMC2872665; doi:10.1371/journal.pone.0010686)
Supplement: Materials and Methods S1 — (0.11 MB DOC) [file pone.0010686.s001.doc]

**Supplementary Information**

# Effect of heterogeneous mixing and vaccination on the dynamics of anthelmintic resistance: a nested model

Lorenzo Sabatelli

Vaccine and Infectious Disease Institute, Fred Hutchinson Cancer Research Center

Seattle, WA, USA,

Email: lsabatel@fhcrc.org

Present address: Institute for Health Metrics and Evaluation

Department of Global Health, University of Washington,

2301 5^th^ Avenue, Suite 600, Seattle WA 98121, USA

Email: sabatell@uw.edu

**Supporting Information Material and Methods S1**

**1) COMPARISON OF MODEL OUTPUTS WITH DATA**

The predictive potential of the model was tested by comparing simulation results with data from published re-infection studies [1,2,3,4]. Re-infection studies are interventional studies in which a population endemically infected, with hookworm in this case, is administered anthelmintic drugs and followed over time. The typical variable of interest is the mean egg count, used as a proxy for the mean worm burden.

The data were obtained from published peer reviewed studies, only studies meeting the following inclusion criteria were considered for comparison with model results:

1. A follow-up period no shorter than 18 months
2. Treatment administered independently of age

These criteria allow only the inclusion of two studies [1,3] out of the four studies initially identified [1,2,3,4].

The simulation was performed using the baseline parameter values reported in table 1 of the main text, with the exception of coverage and endemic worm burden, for Papua New Guinea [1] *W*=25.3, while for India [3] *W*=24.5, *W*=25 is used in the simulation (the results are very little sensitive to W variations within the range 24-26 worms/person). Due to uncertainties about the actual coverage achieved in these studies, values of *c* between 0.1 and 0.4 where used in the simulation.

The results (not displayed) suggest a good agreement between the time-scales of the simulated re-infection process and the time-scales empirically observed.

# 2) ESTIMATION OF THE THRESHOLD FOR TRANSITION FROM FAST TO SLOW REGIME OF RECESSIVE ALLELES.

The threshold for transition from fast to slow regime corresponds to the value of ρ above which the curves in Figure 2A (main paper) exhibit a point of inflexion, becoming convex, and the curve in Figure 2 B (main paper) becomes concave.

This estimate is affected by population size effects and errors associated with numerical approximation, with the actual value lying between 68.5% and 71.2%, if density dependence acts on parasite fecundity (eq. 1.1) and between 63.9%-69.2.% if density dependence acts on establishment (eq. 1.6).

**3) A SIMPLE DETERMINISTIC MODEL FOR THE SPREAD OF RECESSIVE ALLELES**

To disentangle the effect of genetics and mixing from other aspects of the model, such as density dependence and population structure, and understand the origin of the two-regime behavior observed for recessive resistant genes, a simple deterministic model of allele transmission and spread was developed. It consists of only two compartments (A and B).

Both compartments A and B may contain homozygous resistant (RR), heterozygous susceptible (RS) and homozygous susceptible (SS) worms, interacting according to Mendel’s laws.

$$\begin{matrix} RR+RR\to RR \\ RS+RR\to\frac{1}{2}RR+\frac{1}{2}RS\begin{matrix} & \begin{matrix} & \end{matrix} & \end{matrix} \\ RS+RS\to\frac{1}{4}RR+\frac{1}{2}RS+\frac{1}{4}SS\begin{matrix} & \begin{matrix} \begin{matrix} & & \end{matrix} & & \end{matrix} \end{matrix} \\ RS+SS\to\frac{1}{2}RS+\frac{1}{2}SS\begin{matrix} & & & \end{matrix} \\ SS+SS\to SS \end{matrix}$$

(S3.1)

At the beginning of the simulation, homozygous resistant and heterozygous parasites are in compartment A (focus of resistance), and homozygous susceptible parasites are in compartment B. The initial frequency of resistant alleles is 1%, parasites carrying the gene of resistance account for 1.99 % of the parasite population (equal to $p^{2}+2p\left(1-p\right)$as if at Hardy-Weinberg equilibrium). A proportion  of the offspring generated in compartment A remains in compartment A, while a fraction 1- of the offspring flows to compartment B. Like-wise, to ensure, in the absence of interventions, endemic population-equilibrium between the two compartments, a fraction $\pi=\left(p^{2}+2p\left(1-p\right)\right)\left(1-\rho\right)/\left(\left(1-p\right)\left(1-p\right)\right)$ of the offspring generated within the general parasite population will invade the focus.  measures the degree of isolation of compartment A. It can be shown that the invasion of the focus from externally (general population) generated heterozygous and homozygous resistant offspring has no significant impact on the variable of interest: the frequency of resistant genes in the general population. Therefore only the in-flux of homozygous susceptible parasites will be considered (eq. S3.4).

Let$V_{1}$,$V_{2}$, and $V_{3}$ denote the number of within-focus homozygous resistant, heterozygous and homozygous susceptible parasites; $\bar{V}_{1}$, $\bar{V}_{2}$, and $\bar{V}_{3}$ denote the number of homozygous resistant, heterozygous and homozygous susceptible parasites in the general population. Let *c* be the chemotherapy coverage and δ the drug induced parasite-mortality, ρ the degree of host-population mixing $R_{0}$is the basic reproductive rate and μ is the natural mortality rate of parasites.

The model was implemented using ordinary differential equations and reads:

(dynamics of parasites within compartment A, the focus of resistance)

$\frac{d}{dt}V_{1}\left(t\right)=\rho\mu R_{0}\left(\left(V_{1}^{2}+V_{1}V_{2}+\frac{1}{4}V_{2}^{2}\right)/V_{TOT}\right)-\mu V_{1}\begin{matrix} & \begin{matrix} & & & \end{matrix} & & \end{matrix}$ (S3.2)

$\frac{d}{dt}V_{2}\left(t\right)=\rho\mu R_{0}\left(\left(V_{1}V_{2}+2V_{1}V_{3}+V_{2}V_{3}+\frac{1}{2}V_{2}^{2}\right)/V_{TOT}\right)-\mu V_{2}-\delta V_{2}\begin{matrix} & \begin{matrix} & & \end{matrix} & & \end{matrix}$ (S3.3)

$$\frac{d}{dt}V_{3}\left(t\right)=\rho\mu R_{0}\left(\left(V_{3}^{2}+V_{2}V_{3}+\frac{1}{4}V_{2}^{2}\right)/V_{TOT}\right)-\mu V_{3}-\delta V_{3}+\pi\mu R_{0}\left(\left(\bar{V}_{3}^{2}+\bar{V}_{2}\bar{V}_{3}+\frac{1}{4}\bar{V}_{2}^{2}\right)/\bar{V}_{TOT}\right)$$

(S3.4)

(dynamics of parasites in compartment B, representing the general host population, outside the focus of resistance)

$\frac{d}{dt}\bar{V}_{1}\left(t\right)=\mu R_{0}\left(\left(\bar{V}_{1}^{2}+\bar{V}_{1}\bar{V}_{2}+\frac{1}{4}\bar{V}_{2}^{2}\right)/\bar{V}_{TOT}\right)-\mu\bar{V}_{1}+\left(1-\rho\right)\mu R_{0}\left(\left(V_{1}^{2}+V_{1}V_{2}+\frac{1}{4}V_{2}^{2}\right)/V_{TOT}\right)$ (S3.5)

$\frac{d}{dt}\bar{V}_{2}\left(t\right)=\mu R_{0}\left(\left(\bar{V}_{1}\bar{V}_{2}+2\bar{V}_{1}\bar{V}_{3}+\bar{V}_{2}\bar{V}_{3}+\frac{1}{2}\bar{V}_{2}^{2}\right)/\bar{V}_{TOT}\right)-\mu\bar{V}_{2}-c\delta\bar{V}_{2}+\left(1-\rho\right)\mu R_{0}\left(\left(V_{1}V_{2}+2V_{1}V_{3}+V_{2}V_{3}+\frac{1}{2}V_{2}^{2}\right)/V_{TOT}\right)$ (S3.6)

$\frac{d}{dt}\bar{V}_{3}\left(t\right)=\left(1-\pi\right)\mu R_{0}\left(\left(\bar{V}_{3}^{2}+\bar{V}_{2}\bar{V}_{3}+\frac{1}{4}\bar{V}_{2}^{2}\right)/\bar{V}_{TOT}\right)-\mu\bar{V}_{3}-c\delta\bar{V}_{3}+\left(1-\rho\right)\mu R_{0}\left(\left(V_{3}^{2}+V_{2}V_{3}+\frac{1}{4}V_{2}^{2}\right)/V_{TOT}\right)$ (S3.7)

The parameters are chosen according to table 1. The model output is studied for δ between zero and 1.6. It is important to notice that, unlike the main model, in the deterministic model drug-induced parasite removal is a continuous process occurring at a rate .

The factors:

$$\left(V_{1}^{2}+V_{1}V_{2}+\frac{1}{4}V_{2}^{2}\right)/V_{TOT}$$

$$\left(V_{1}V_{2}+2V_{1}V_{3}+V_{2}V_{3}+\frac{1}{2}V_{2}^{2}\right)/V_{TOT}$$

$$\left(V_{3}^{2}+V_{2}V_{3}+\frac{1}{4}V_{2}^{2}\right)/V_{TOT}$$

weigh the production of homozygous resistant, heterozygous and homozygous susceptible parasites according to Mendel’s laws [5], assuming an equal number of male and female parasites.

The quantity$FA=\left(2\bar{V}_{1}+\bar{V}_{2}\right)/2\bar{V}_{TOT}$ represents the frequency of resistant alleles in the general population (compartment B). The dynamics of *FA*, in Figure S3, shows that the selective pressure exerted by anthelmintics is instrumental to determining a two-regime dynamical behavior. If <0.35 *FA* decreases monotonically with ρ and decreases thereafter. If >0.35, depending on ρ, there are two alternative dynamical regimes associated with the spread of resistant alleles outside the primary focus of resistance, depending on whether the proportion of within-focus host-to-host transmission ρ, which is associated with parasite inbreeding, in the initial focus of resistance, is below or above a “threshold value” of the allele frequency in the parasite population: 1) undergoes a fast growth for 2-5 years and then a much (about 10 times) slower growth for the following 80 years, or 2) grows slowly for the first 8-12 years and then rises fast to 1 (strain replacement) and plateaus. For very small initial frequencies *p*<1/100000 regime (1) leads to an actual plateau with a limit value much smaller than 1.

A non-monotonic relationship between the allele frequency reached after a period of time *T*=20 years and  is observed.

The qualitative results of the deterministic model are robust to univariate changes of key parameters such as the basic reproductive number$R_{0}$(i.e. 2.5-3.5), the natural parasite mortality rate μ (i.e. 0.15-0.25/year), the endemic mean worm burden *W* (i.e. 10-30 worms/person), the initial prevalence of resistant genes *p* (i.e. 0.01%-4%), the intervention-coverage *c* (i.e. 30%-60%) and the time horizon of the simulation (i.e. 15-35 years).

This deterministic model was implemented with Berkeley Madonna (version 8.3.11) and the code is here provided to the benefit of readers willing to explore this topic further.

#### Deterministic model code for Berkeley-Madonna software (version 8.3.11):

**METHOD RK4**

**STARTTIME = 0**

**STOPTIME=20**

**DT = 0.02**

**d/dt(V1)=rho*mu*R0*((V1*V1+V1*V2+0.25*V2*V2)/(V1+V2+V3))-mu*V1**

**d/dt(V2)=rho*mu*R0*((V1*V2+2*V1*V3+V2*V3+0.5*V2*V2)/(V1+V2+V3))-dr*V2-mu*V2**

**d/dt(V3)=rho*mu*R0*((V3*V3+V3*V2+0.25*V2*V2)/(V1+V2+V3))-dr*V3-mu*V3+ pp*mu*R0*((V3ext*V3ext+V3ext*V2ext+0.25*V2ext*V2ext)/(V1ext+V2ext+V3ext))**

**d/dt(V1ext)=mu*R0*((V1ext*V1ext+V1ext*V2ext+0.25*V2ext*V2ext)/(V1ext+V2ext+V3ext))-mu*V1ext+((1-rho)*mu*R0*((V1*V1+V1*V2+0.25*V2*V2)/(V1+V2+V3)))**

**d/dt(V2ext)=mu*R0*((V1ext*V2ext+2*V1ext*V3ext+V2ext*V3ext+0.5*V2ext*V2ext)/(V1ext+V2ext+V3ext))-dr*cov*V2ext-mu*V2ext+((1-rho)* mu*R0*((V1*V2+2*V1*V3+V2*V3+0.5*V2*V2)/(V1+V2+V3)) )**

**d/dt(V3ext)=(1-pp)*mu*R0*((V3ext*V3ext+V3ext*V2ext+0.25*V2ext*V2ext)/(V1ext+V2ext+V3ext))-dr*cov*V3ext-mu*V3ext +((1-rho)*mu*R0*((V3*V3+V3*V2+0.25*V2*V2)/(V1+V2+V3)) )**

**Vext=V1ext+V2ext+V3ext**

**V=V1+V2+V3**

**VTOT=V+Vext**

**F=(2*V1ext+V2ext)**

**FA=F/(2*Vext)**

**mu=0.2**

**R0=3**

**rho=0**

**p=0.01**

**pp=((p*p+2*p*(1-p))*(1-rho))/((1-p)*(1-p))**

**N=10000**

**W=20**

**dr=0.8**

**cov=0.5**

**init V1=N*W*p*p**

**init V2=2*N*W*p*(1-p)**

**init V3=0**

**init V1ext=0**

**init V2ext=0**

**init V3ext=N*W*(1-p)*(1-p)**

**{V1=homozygous resistant; V2=heterozygous susceptible; V3= homozygous susceptible}**

**{V1ext=external homozygous resistant; V2ext=external heterozygous susceptible; V3ext= external homozygous susceptible}**

**{mu=adult worm mortality rate; R0=basic reproductive number; }**

**{rho= proportion of the offspring produced within the focus that stays in the focus}**

**{p=initial frequency of resistant alleles; N= host-population size; W=mean worm burden;}**

**{dr=drug-induced mortality rate; cov= drug coverage}**

**{FA is the frequency of resistant alleles outside the focus of resistance}**

#### References

1. Quinnell RJ, Slater AF, Tighe P, Walsh EA, Keymer AE, et al. (1993) Reinfection with hookworm after chemotherapy in Papua New Guinea. Parasitology 106: 379-385.

2. Albonico M, Smith P, Ercole E, Hall A, Chwaya H, et al. (1995) Rate of reinfection with intestinal nematodes after treatment of children with mebendazole or albendazole in a highly endemic area. Trans R Soc Trop Med Hyg 89: 538-541.

3. Schad GA, Anderson RM (1985) Predisposition to hookworm infection in humans. Science 228: 1537-1540.

4. Shield JM, Vaterlaws AL, Kimber RJ, Payne R, Casey GJ, et al. (1981) The relationship of hookworm infection, anaemia and iron status in a Papua New Guinea highland population and the response to treatment with iron and mebendazole. P N G Med J 24: 19-34.

5. Hartwell LH, Hood, L.,Goldberg, M.L.,Reynolds, A.E.,Silver L.M.,Veres, R. C. (2008) Genetics: from Genes to Genome. New York: McGrawHill.
