# Supplementary figures and images for: Effect of Heterogeneous Mixing and Vaccination on the Dynamics of Anthelmintic Resistance: A Nested Model
Source: PLoS One. 2010 May 18;5(5):e10686. doi: 10.1371/journal.pone.0010686 (PMC2872665; doi:10.1371/journal.pone.0010686)

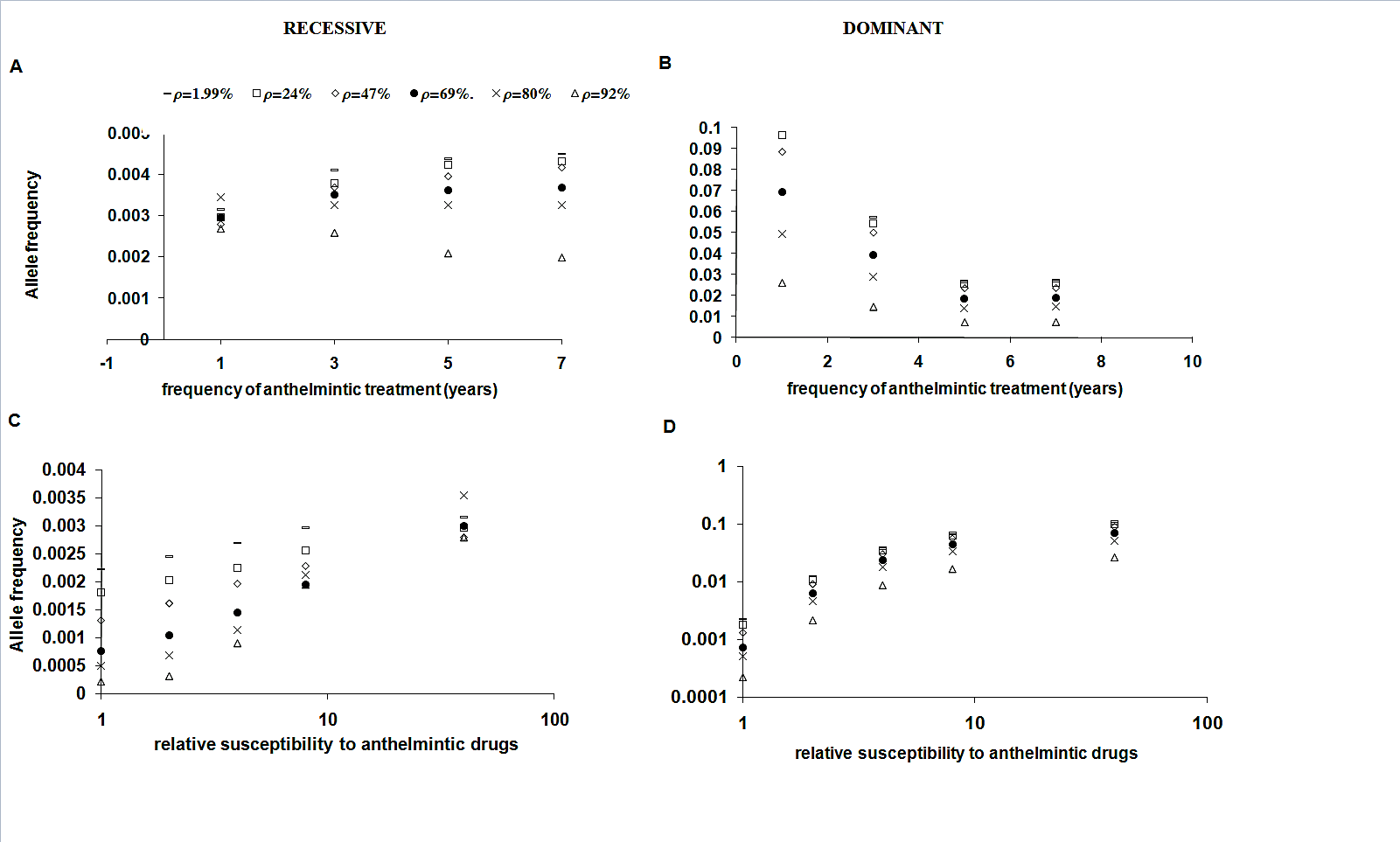

Supplement: Figure S1 — Frequency of resistant alleles after 10 years of chemotherapy. A) Reducing the frequency of treatment does not reduce the frequency of recessive alleles, unless the focus of resistance is highly isolated (ρ = 92%). B) Reducing the frequency of treatment always reduces the frequency of dominant alleles. The frequencies of (C) recessive and (D) dominant alleles increase with the relative susceptibility to drugs, defined as the ratio of the probabilities of being killed by drugs for susceptible and resistant worms. Baseline parameters are in Table 1 of the main paper. (4.92 MB TIF) [file pone.0010686.s002.tif]

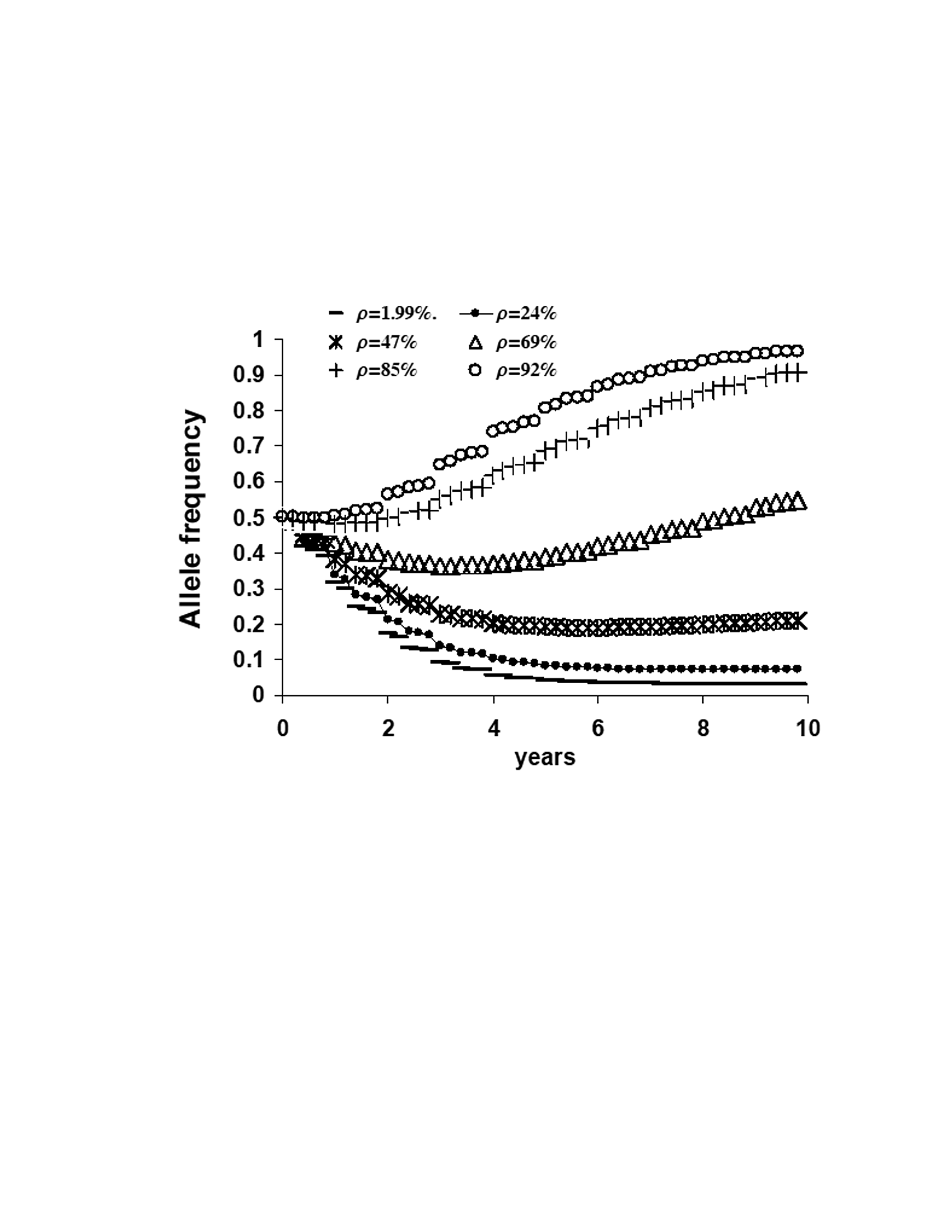

Supplement: Figure S2 — Frequency of recessive alleles within the focus. (4.03 MB TIF) [file pone.0010686.s003.tif]

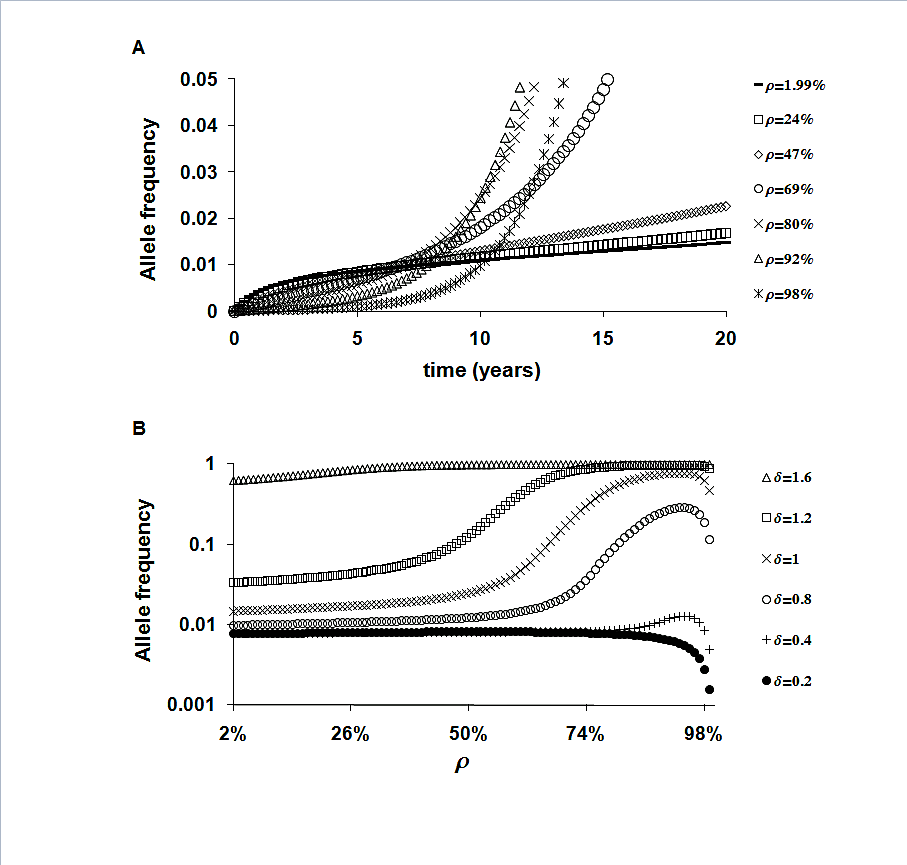

Supplement: Figure S3 — Results from a simple deterministic model. A) If the anthelmintic associated rate of death is higher than 0.35 the allele frequency follows two different time-dynamics depending on the degree of host-mixing ρ: i) it grows fast and then very slow ii) a slow initial growth is followed by an accelerated growth. B) Non-monotonic relation between the allele frequency after 20 annual chemotherapy rounds and ρ obtained for different values of δ. The difference in allele frequency between the two dynamical regimes decreases as the selection-pressure-related parameter δ increases. Baseline parameters are in Table 1 of the main paper. (2.61 MB TIF) [file pone.0010686.s004.tif]

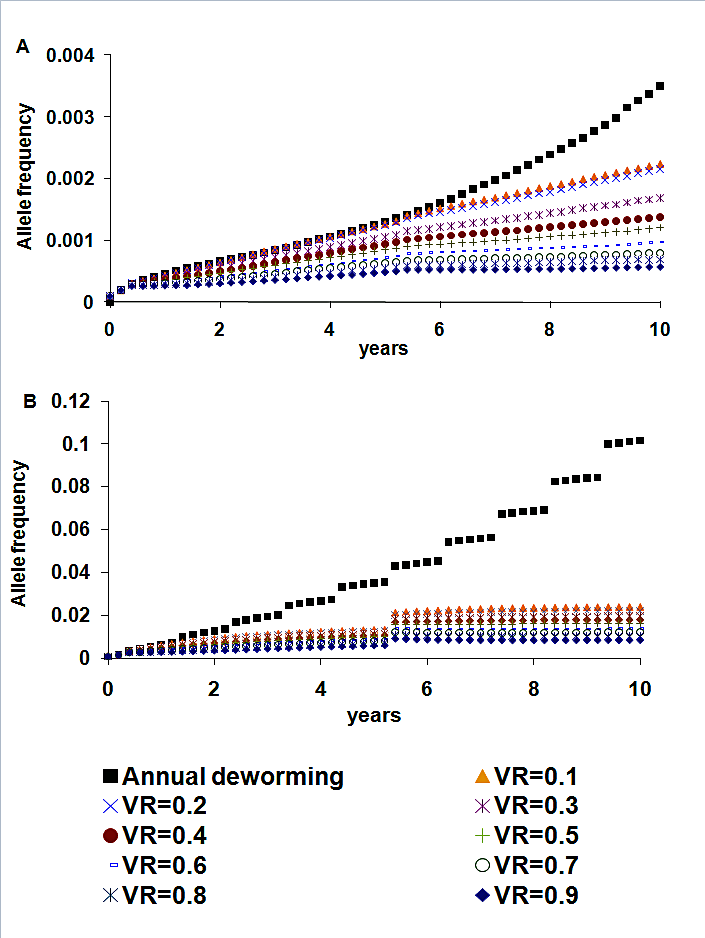

Supplement: Figure S4 — Effect of vaccination on the dynamics of drug resistance. In this example the vaccine reduces by a proportion VR the average reproductive rate of female parasites. A) Recessive case: the reduction in the spread of recessive resistant alleles primarily depends on the efficacy of the vaccine (in this example, the vaccine acts on female worm fecundity and ρ = 85%). B) Dominant case: the reduction in the spread of dominant recessive alleles primarily depends on the reduction of the frequency of treatment (in this example, every 5 years instead of every year). Baseline parameters are in Table 1 of the main paper. (2.23 MB TIF) [file pone.0010686.s005.tif]

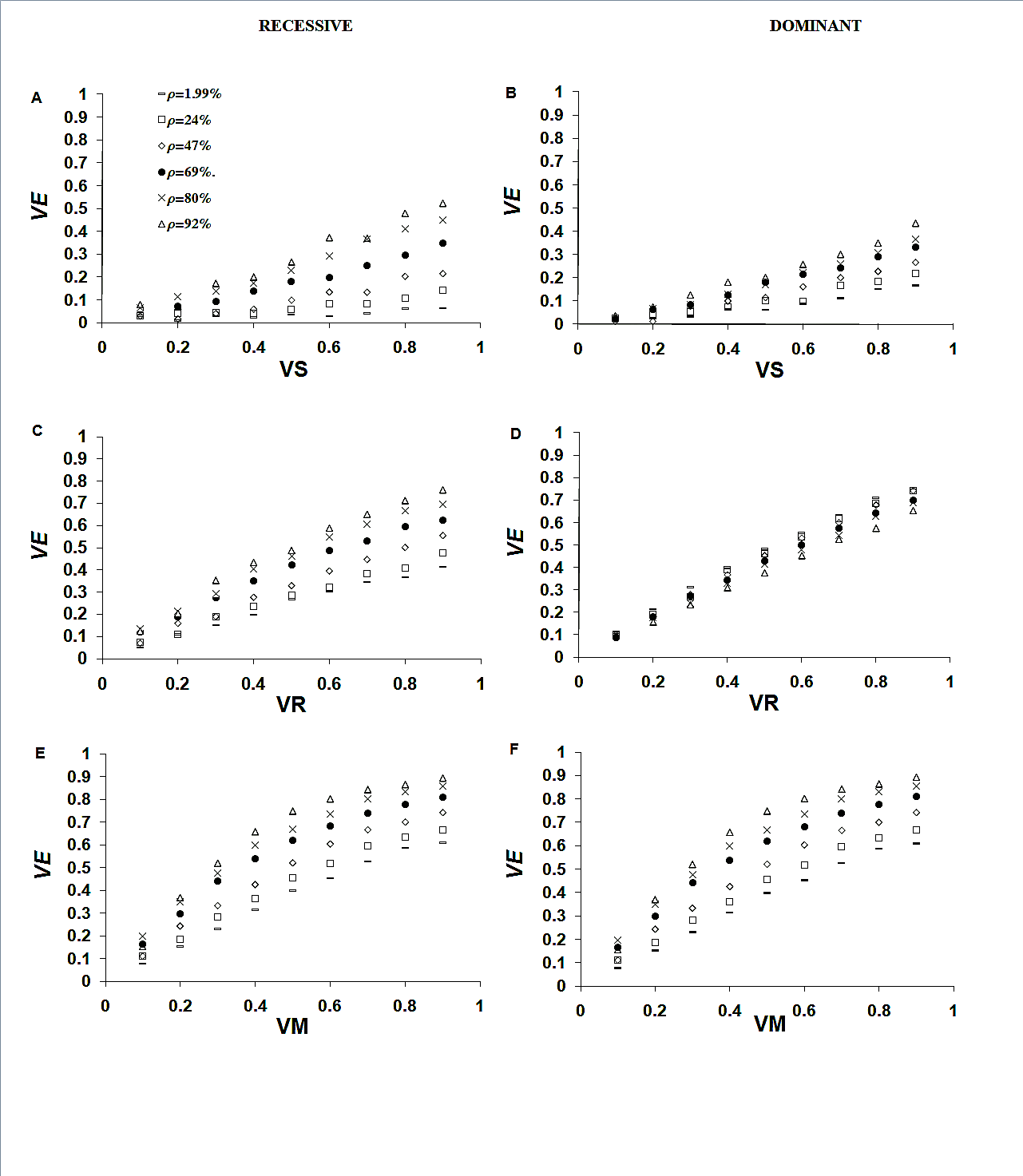

Supplement: Figure S5 — Vaccine effectiveness (VE) when density dependence acts on the establishment of new worms. Vaccines can reduce the host susceptibility to infection (A–B), by a proportion VS, or the female parasite fecundity (C–D), by a proportion VR, or the parasite lifespan (E–F), or by a proportion VM. Vaccines reducing the parasite fecundity and vaccines reducing the parasite lifespan are more efficacious than vaccines reducing host susceptibility by the same proportion. (6.01 MB TIF) [file pone.0010686.s006.tif]
