# Supplementary material for: Effect of Heterogeneous Mixing and Vaccination on the Dynamics of Anthelmintic Resistance: A Nested Model
Source: PLoS One. 2010 May 18;5(5):e10686. doi: 10.1371/journal.pone.0010686 (PMC2872665; doi:10.1371/journal.pone.0010686)
Supplement: Table S1 — Univariate sensitivity analysis: Threshold for transition ρ from fast to slow dynamics of recessive alleles. The value of ρ, estimated at baseline, is 69%. Intervals refer to maximum and minimum ρ values. Impact of a vaccine reducing host susceptibility for VS = 50% and ρ. Intervals of maximum and minimum percentage variation of vaccine impact VE, from baseline values VE = 18% (recessive) and VE = 17% (dominant) observed when parameter values vary within the given ranges. Baseline parameters are in Table 1 of the main paper. (0.04 MB DOC) [file pone.0010686.s007.doc]

| **Parameter** | **Range** | **ρ interval** | **Vaccine effectiveness** | |
| --- | --- | --- | --- | --- |
|  |  |  | **Recessive** | **Dominant** |
| $\alpha_{g,sus}$ | 70-90% | 67%-73% | -32% ; 3% | -25%-0% |
| $\alpha_{g,res}$ | 1-3% | 60%-76% | -51%; 4% | -34%-0% |
| $R_{o}$ | 2.5-3.5 | 62%-73% | -19% ; +28% | -11%; +20% |
| c | 0.4-0.6 | 64%-74% | -27%; +23% | -22%; +20% |
|  μ | 0.15-0.25/year | 61%-78% | -9%; +12% | -8%; +10% |
| *W* | 18-22 worms/person | 66%-74% | -7%; +8% | -5%; +9% |
| *k* | 0.3-0.0.38 | 62%-77% | -28%; +39% | -20%; +29% |
| **Ω** | 0-0.002 | 65%-76% | 0; 8% | -3%; 0 |
| **** | 0-0.1/year | 0* | 0* | 0* |

*Because at baseline ****
