# Supplementary material for: Effect of Heterogeneous Mixing and Vaccination on the Dynamics of Anthelmintic Resistance: A Nested Model
Source: PLoS One. 2010 May 18;5(5):e10686. doi: 10.1371/journal.pone.0010686 (PMC2872665; doi:10.1371/journal.pone.0010686)
Supplement: Table S2 — Multivariate Sensitivity Analysis: Variation of the threshold for transition ρfrom fast to slow dynamics of recessive alleles. The value of ρ, estimated at baseline, is 69%. The density-dependent parameters are chosen to fit the endemic equilibrium data. Baseline parameters are in Table 1 of the main paper. (0.04 MB DOC) [file pone.0010686.s008.doc]

| **Parameters changed** | **Range** | **Minimum**  **** | **Maximum**  ** ** |
| --- | --- | --- | --- |
| $R_{o}$; *k* | 2-6 ; 0.2-0.5 | 50% | 87% |
| $R_{o}$*;* c | 2-6; 0.25-0.9 | 59% | 78% |
| ; Ω | 0-3y; 0-0.003 | 63% | 79% |
|  | 1-100 | >66% | no-transition |
| *W* | 10-25 | 62% | 81% |
