# Supplementary material for: Effect of Heterogeneous Mixing and Vaccination on the Dynamics of Anthelmintic Resistance: A Nested Model
Source: PLoS One. 2010 May 18;5(5):e10686. doi: 10.1371/journal.pone.0010686 (PMC2872665; doi:10.1371/journal.pone.0010686)
Supplement: Table S3 — Sensitivity analysis of the impact of a vaccine reducing host susceptibility for VS = 50% and ρ = 69%. Intervals of percentage variation of vaccine impact VE, from baseline values VE = 18% (recessive) and VE = 17% (dominant) observed when parameter values vary within the given ranges. The density-dependence parameters are chosen to fit the endemic mean worm burden W Min and Max are the minimal and maximal percentage deviation from the simulation results obtained using baseline parameters, when density-dependent regulatory mechanisms act on parasite fecundity. Baseline parameters are in Table 1 of the main paper. (0.04 MB DOC) [file pone.0010686.s009.doc]

| **Parameters changed** | **Range** | **Recessive** | | **Dominant** | |
| --- | --- | --- | --- | --- | --- |
|  |  | **min** | **Max** | **min** | **Max** |
| $R_{o}$; *k* | 2-6; 0.2-0.5 | -54% | +67% | -45% | +59% |
| $R_{o}$; c | 2-6; 0. 25-0.9 | -43% | +99% | -23% | +54% |
|  Ω | 0-3y ; 0-0.003 | 0% | +10% | -3.4% | 0% |
|  | 1-100 | -100% | 0% | -100% | 0% |
| *W* | 10-25 | -7% | +8% | -5% | +9% |
