# Supplementary material for: Effect of Heterogeneous Mixing and Vaccination on the Dynamics of Anthelmintic Resistance: A Nested Model
Source: PLoS One. 2010 May 18;5(5):e10686. doi: 10.1371/journal.pone.0010686 (PMC2872665; doi:10.1371/journal.pone.0010686)
Supplement: Table S4 — Sensitivity Analysis of the impact of a vaccine reducing female parasite fecundity, for VR = 50% and ρ = 69%. Intervals of percentage variation of vaccine impact VE, from baseline values VE = 18% (recessive) and VE = 17% (dominant) observed when parameter values vary within the given ranges. The density-dependence parameters are chosen to fit the endemic mean worm burden W. Min and Max are the minimal and maximal percentage deviation from the simulation results obtained using baseline parameters, when density-dependent regulatory mechanisms act on parasite fecundity. Baseline parameters are in Table 1 of the main paper. (0.05 MB DOC) [file pone.0010686.s010.doc]

| **Parameters changed** | **Range** | **Recessive** | | **Dominant** | |
| --- | --- | --- | --- | --- | --- |
|  |  | **min** | **Max** | **min** | **Max** |
| $R_{o}$; *k* | 2-6; 0.2-0.5 | -55% | +65% | -44% | +51% |
| $R_{o}$; *c* | 2-6; 0.25-0.9 | -15% | +19% | -13% | +17% |
|  Ω | 0-3y; 0-0.003 | 0% | +2% | 0% | +0.8% |
|  | 1-100 | -100% | 0% | -100% | 0% |
| *W* | 10-25 | -6.6% | +8.5% | -5.2% | +9% |
